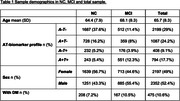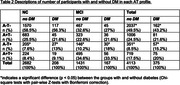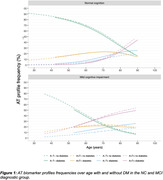# The association of Diabetes Mellitus with Amyloid and Tau biomarkers

**DOI:** 10.1002/alz.091311

**Published:** 2025-01-09

**Authors:** Stephan Duijkers, Jolien van der Velden, Veerle van Gils, Julie Elisabeth Oomens, Stephanie J. B. Vos, Inez H.G.B. Ramakers, Pieter Jelle Visser, Willemijn J. Jansen, the Amyloid Biomarker Study group

**Affiliations:** ^1^ Alzheimer Center Limburg, School for Mental Health and Neuroscience, Maastricht University, Maastricht Netherlands; ^2^ Maastricht Universy Medical Centre, Maastricht Netherlands; ^3^ Alzheimer Center and Department of Neurology, Amsterdam Neuroscience Campus, VU University Medical Center, Amsterdam Netherlands

## Abstract

**Background:**

As global populations age, both Alzheimer’s Disease (AD) and diabetes mellitus (DM) incidence are projected to increase tremendously over the coming 20‐30 years. Studies have found that having DM is associated with increased risk of developing AD dementia. It remains unclear however whether DM impacts underlying AD pathology. In the current study we investigate associations between DM and profiles of amyloid‐β‐42 (A) and phosphorylated tau‐181 (T) pathology in cerebrospinal fluid (CSF) in participants with normal cognition (NC) and mild cognitive impairment (MCI).

**Methods:**

We included 2890 participants with NC and 1598 participants with MCI from 21 studies of the Amyloid Biomarkers Study. Participants were classified into four AT biomarker profiles: A‐T‐, A+T‐, A+T+ and A‐T+, based on data‐driven or cohort‐specific cutoffs. All participants had data on DM diagnosis (yes/no). Bonferroni corrected chi‐square tests with pair‐wise Z‐tests were performed to compare the observed AT biomarker profile frequency across DM groups. To examine whether age‐related changes in AT biomarker profile frequencies were dependent on DM, an interaction between age and DM was entered into a Markov‐Chain‐Monte‐Carlo generalized linear mixed model.

**Results:**

Table 1 displays sample demographics. Table 2 displays differences between participants with and without DM per AT profile. In both NC and MCI, participants with DM more often had the A‐T+ biomarker profile than participants without DM (NC: 7.6% vs. 13%; MCI: 10.2% vs. 18%; both p<0.05), while frequencies of the A‐T‐, A+T‐ and A+T+ biomarker profiles were similar among DM groups. In both NC and MCI, DM status did not impact the age‐related increase in AT biomarker frequencies (NC p = 0.34; MCI p=0.45) (Figure 1).

**Conclusions:**

These results demonstrate that for both NC and MCI, persons with DM more often have an A‐T+ profile compared to those without diabetes, suggesting that people with DM have an increased risk of isolated tau pathology that may be independent from amyloid pathology. DM did not change frequencies of biomarker profiles across age. These findings warrant further research and could have implications for both diagnostics and clinical trial design.